# Time Effects of Supportive Interaction and Facilitator Input Variety on Treatment Adherence of Young People with Chronic Health Conditions: A Dynamic Mechanism in Mutual Aid Groups

**DOI:** 10.3390/ijerph18063061

**Published:** 2021-03-16

**Authors:** Steven Sek-Yum Ngai, Chau-Kiu Cheung, Yuen-Hang Ng, Liang Shang, Hon-Yin Tang, Hiu-Lam Ngai, Kenix Hok-Ching Wong

**Affiliations:** 1 Department of Social Work, The Chinese University of Hong Kong, Hong Kong, China; yhng@swk.cuhk.edu.hk (Y.-H.N.); echoshang@cuhk.edu.hk (L.S.); hytang@cuhk.edu.hk (H.-Y.T.); ngaihiulam@link.cuhk.edu.hk (H.-L.N.); hokchingwong92@gmail.com (K.H.-C.W.); 2 Department of Social and Behavioral Sciences, City University of Hong Kong, Hong Kong, China; ssjacky@cityu.edu.hk

**Keywords:** mutual aid group, treatment adherence, supportive interaction, facilitator input, time effects, young patients

## Abstract

This study aims to examine the mechanism of how supportive interaction and facilitator input variety in mutual aid groups impact treatment adherence of young people with chronic health conditions, with consideration of time effects, which have been rarely studied in the existing literature. A stratified random sample of 391 individuals aged 12–45 years with chronic health conditions were recruited from mutual aid groups in Hong Kong and completed both the baseline and 12-month follow-up surveys. The results of the structural equation modeling indicated that supportive interaction and facilitator input variety positively predicted treatment adherence in a delayed condition, whereas members’ treatment adherence in the baseline survey had reversed effects on members’ supportive interaction in the follow-up survey. The findings of this study shed light on the dynamic mechanism of the mutual aid groups and provide important implications to promote better rehabilitation outcomes of young people with chronic health conditions.

## 1. Introduction

Chronic health conditions, which are defined as recurrent illnesses lasting for one year or more, such as mental illnesses, asthma, diabetes, and heart disease [1], have been a complex challenge to the global medical system for the rapidly increasing patient population. From 1990 to 2017, the number of people with chronic health conditions (PCHC) increased by 40% [2], while the early onset of chronic health conditions has become a prevalent issue among young people [3]. Young PCHC face many personal challenges in daily life, including demanding treatment regiments, treatment side effects, and in some cases, the prospect of a shortened future [4]. For young PCHC, such challenges have been intensified in a broader social and environmental context concerning issues like educational accessibility and career development [3]. Treatment adherence, which refers to the regularity with which a patient follows treatment plans advised by healthcare professionals, has been seemingly recognized as vital to achieving successful rehabilitation outcomes [5]. Notably, sufficient treatment adherence could enhance the quality of life and reduce morbidity and mortality from diseases, further alleviating the medical and financial burdens at personal and societal levels [6].

The extant literature suggests that mutual aid groups play an important role in enhancing and maintaining treatment adherence of group participants (e.g., [7]). A mutual aid group has been commonly defined as a group of people sharing a similar problem, who meet regularly to exchange knowledge and information and to give and receive emotional and psychological support [8]. In particular, the members of the group support one another by providing emotional support, exchanging information, sharing experiences, and assisting in problem solving [9]. The effectiveness of the group not only depends on the supportive actions from members, but also on facilitators playing an important role in the group, contributing their knowledge and skills as well as encouraging hope [9]. Mutual aid groups cover topics such as illness management, problem-solving, and emotion regulation [10]. Such participation was found to be associated with many rehabilitation outcomes, such as better mental well-being [11], reduced depressive symptoms [12], improved management of illness [13], and better treatment adherence [14]. Importantly, according to the literature, while the mutual aid group approach is cost-effective and sustainable as it mainly leverages peer support without demanding much professional involvement [7], it is also a helping process of relatively longer duration since its success depends on the mutual trust and reciprocal relationships among group members [8]. This implies that such an approach may take time for the participation to create an impact. Although many studies have already pointed out the types of changes that members experience in mutual aid groups, these studies were not designed to investigate relationships between influential elements of mutual aid groups and rehabilitation outcomes, such as treatment adherence of members (e.g., [13]). Given this research gap, the current study aims to explore the dynamic mechanism of mutual aid groups and uncover the relationships between important components of mutual aid groups and treatment adherence of young PCHC from a time perspective.

### 1.1. Supportive Interaction, Facilitator Input Variety, and Treatment Adherence

Among various components of mutual aid groups, supportive interaction and facilitator input variety appear to be influential to treatment adherence of the group members (e.g., [15,16]). Supportive interaction, which refers to the communication of feelings and exchange of mutual support and experiential knowledge among group members, tends to have a positive influence on treatment adherence of the members [14,17]. Mutual aid groups in particular can be conceptualized as drawing on the potential benefits of socially supportive interactions, since they utilize support from people who have gone through similar difficulties so that participants can easily empathize with each other [13]. Through supportive interaction, young PCHC potentially get emotional assistance and information that may be beneficial to their chronic illness management. As members of mutual aid groups unite to understand others’ needs and concerns, give thoughtful responses, and share their experiences, they may feel more understood and less isolated [17], develop a sense of empowerment and self-efficacy [18], acquire useful knowledge and skills to cope with the chronic conditions [14], and gain self-worth when they contribute to the groups [19]. In this process, group members are encouraged to acquire more effective ways of coping with one’s difficulties and thus, in turn, improve their treatment adherence [15,18].

Facilitator input refers to assistance offered by a mutual aid group facilitator in helping group members to consider and address problems faced by the members [20]. The facilitators may be helping professionals, such as social workers or allied health workers, or non-professional persons, such as ex-patients who have gone through chronic health conditions with good illness management [21]. Moreover, facilitator input in mutual aid groups may cover a wide range of topics, including work, education, finance, emotional and behavioral problems, physical health, and family [22,23]. Importantly, a high variety of facilitator input implies the adoption of a holistic and multidimensional approach by the facilitator, rather than using a unidimensional approach in assisting PCHC [24]. Such a holistic approach adopted by the mutual aid group facilitator is necessary as chronic health conditions can have detrimental effects on various areas of people’s lives, such as decreasing people’s ability to work [25], causing emotional and behavioral problems [22], and straining relationships with family and friends [23]—all of which may lead to poor treatment adherence. Existing studies have already pointed out that based on the topics discussed in mutual aid groups, facilitator input could trigger a positive influence on many different aspects of PCHC’s lives such as physical health [15], family relationships [21], and emotion and behavior management (e.g., [26]). As chronic illness-related problems are multidimensional [27], a more comprehensive input by the facilitator in mutual aid groups could potentially lead to better improvements in the treatment adherence of group members.

### 1.2. Time Effects of Supportive Interaction and Facilitator Input Variety

However, despite the importance of supportive interaction and facilitator input variety in influencing treatment adherence of PCHC, the mechanism of such influences has been underexplored, especially in the context of young PCHC. Importantly, time appears to be a vital influential factor to mutual aid groups, as it takes time for group members to get to know and care about one another, and it also takes time for group facilitators to help members translate the group’s vision into useful, real-world action [28]. Steinberg [29] pointed out that it was unrealistic to expect mutual aid groups to have the capacity to produce an instant impact as group members need time to establish trust and safety. As such, supportive interaction and facilitator input variety in mutual aid groups may need time to develop impact [18], implying that the evaluation of mutual aid groups needs to be sensitive to potential time effects.

Accordingly, this study aims at exploring the underlying mechanism of how supportive interaction and facilitator input variety impact treatment adherence of young PCHC with consideration of potential time effects. This study identifies three different types of time effects in the mutual aid group settings, including delayed effects, immediate effects, and reversed effects. A delayed effect refers to a change in treatment adherence behaviors of mutual aid group members that can only be measured after a certain time (e.g., 12 months). Indeed, effective implementation of mutual aid groups is based on mutual trust and shared interests among group members [30]. Extant studies have found that mutual aid group members may lack trust and be reluctant to be a part of the group at the beginning [28]. As it takes time for members to build trust and commit themselves to group meetings, some beneficial effects are not apparent until considerable time has elapsed. This time lag may prevent group members from benefiting from the supportive interaction and facilitator input variety immediately after attending the group meetings [26]. Thus, this study hypothesized that supportive interaction and facilitator input variety of mutual aid groups at an earlier time may affect young PCHC’s treatment adherence later. Hence, the following hypotheses concerning the delayed effects were proposed:

**Hypothesis 1 (H1).**  *Young PCHC with a higher level of supportive interaction in mutual aid groups at an earlier time are more likely to have better treatment adherence at a later time.*

**Hypothesis 2 (H2).**  *Young PCHC receiving a wider variety of facilitator input in mutual aid groups at an earlier time are more likely to have better treatment adherence at a later time.*

Next, an immediate effect refers to an instant change of the group member after their mutual aid group meetings. Although this study mainly focuses on examining the delayed effects of supportive interaction and facilitator input variety in mutual aid groups, we were also curious about whether these two influential components had immediate effects on the treatment adherence of group members or not. Therefore, the following hypotheses concerning the immediate effects emerged:

**Hypothesis 3 (H3).**  *Young PCHC with a higher level of supportive interaction in mutual aid groups are more likely to have instant improvements in their treatment adherence.*

**Hypothesis 4 (H4).**  *Young PCHC receiving a wider variety of facilitator input in mutual aid groups are more likely to have instant improvements in their treatment adherence.*

Third, levels of treatment adherence of young PCHC at an earlier time may have reversed effects on supportive interaction and facilitator input variety at a later time, as young PCHC with better treatment adherence tend to be in better physical condition and have higher motivation and energy [31], allowing them to participate in mutual aid groups more actively and reach out for support from facilitators to work on other issues being affected by their chronic health conditions [32]. Importantly, people with better treatment adherence were found to be associated with some attributes, such as having a positive belief toward medications [33], better knowledge about chronic illness management [34], and strong problem-solving skills to manage daily barriers to self-care [35], which are all vital to successful rehabilitation. Consequently, it is reasonable to expect that young PCHC with better treatment adherence may be more able to commit themselves in group interaction, contributing to others’ problems with constructive input to the groups, and actively interacting with facilitators and seeking advice on a variety of areas of their lives. Thus, these hypotheses considering reversed effects were developed:

**Hypothesis 5 (H5).**  *Young PCHC with better treatment adherence at an earlier time are more likely to have higher levels of supportive interaction in mutual aid groups at a later time.*

**Hypothesis 6 (H6).**  *Young PCHC with better treatment adherence at an earlier time are more likely to receive a wider variety of facilitator input in mutual aid groups at a later time.*

Lastly, we were also interested in knowing how supportive interaction and facilitator input variety—two influential components in mutual aid groups—may influence each other at different time points. For example, we wanted to see whether participants receiving more diverse input from facilitators would engage in supportive interaction with group members more actively later in group meetings. Accordingly, this study hypothesized that:

**Hypothesis 7 (H7).**  *Young PCHC with a higher level of supportive interaction in mutual aid groups at an earlier time are more likely to receive a wider variety of facilitator input at a later time.*

**Hypothesis 8 (H8).**  *Young PCHC receiving a wider variety of facilitator input in mutual aid groups at an earlier time are more likely to have a higher level of supportive interaction at a later time.*

## 2. Materials and Methods

### 2.1. Procedure

This panel study collected two waves of data to examine relationships among supportive interaction, facilitator input variety, and treatment adherence of young PCHC from a time perspective, with an average 12-month time interval between the baseline survey and the follow-up survey [36]. Participants were recruited between 2017 and 2018, and their follow-up data were collected between 2018 and 2019. Specifically, a full list of mutual aid groups for PCHC was acquired with the aid of a major non-governmental organization that focuses on supporting persons with disabilities or health challenges in Hong Kong. A stratified random sampling method was adopted, in which the mutual aid groups for PCHC were further divided into ten prevalent chronic illness categories, consisting of asthma, heart disease, diabetes, rheumatic diseases, neurological diseases, hematologic diseases, cancer, rare diseases, eczema, and mental illnesses [37]. In total, 50 mutual aid groups in Hong Kong were selected, with 5 groups from each of these chronic illness categories. The surveys proceeded at service centers where mutual aid groups are held.

All 12- to 45-year young PCHC pertaining to the selected mutual aid groups were included in this study. The participants were notified of the objectives and data-collection procedure of this study. For those aged under 18, parental informed consent was acquired as well. An ethical review committee had evaluated and approved this method prior to administration. A total of 497 young PCHC were recruited in the baseline survey, of whom 391 participated in the follow-up survey. As such, the attrition rate was low and the retention rate was high (approximately 80%). Moreover, the attrition was unrelated to variables involved in the study, according to logistic regression analysis of follow-up response versus attrition (Cox & Snell *R*^2^ = 0.011).

### 2.2. Participants

As shown in Table 1, 391 participants in this study were involved in both baseline and follow-up surveys. Power analysis has shown that the sample of 391 cases should be able to detect a weak effect size of 0.142 with a confidence of 95% and a statistical power of 80%. The detectable effect size is low enough to cover practically meaningful effects. Slightly over half of the participants (55.1%) were female. Most of the participants (75.9%) had a monthly family income of HK$30,000 to 39,999 (US$3871–5160) or below. The educational level of the participants ranged from primary to university education, with 96.5% of them having completed secondary school (grades 7–12) or above. The most frequently reported illness category among the participants was mental illnesses (23.0%), followed by eczema (18.8%), rheumatic diseases (17.5%), neurological diseases (10.7%), hematologic diseases (6.5%), diabetes (5.5%), cancer (5.2%), asthma (5.0%), rare diseases (4.7%), and heart disease (3.1%). The mean age of participants was 30.2 years (*SD* = 7.7). On average, a mutual aid group had 1.6 professional facilitators (*SD* = 2.0) and 0.9 nonprofessional facilitators (*SD* = 1.9).

### 2.3. Measurement

The present study involved a survey design with data collected through a structured questionnaire completed by each participant. The structured questionnaire consisted of the items adapted from the existing literature to measure the predictor variables (i.e., supportive interaction and facilitator input variety) and the outcome variable (i.e., treatment adherence) in mutual aid groups [21,38,39,40]. All items were measured on a five-point scale (1 = never or rarely; 2 = seldom; 3 = average; 4 = quite often; 5 = very often). To facilitate data interpretation and comparison, all the scoring ranged from 0–100 [41].

Treatment adherence refers to the regularity with which a patient follows the treatment plans advised by healthcare professionals [42]. The six-item treatment adherence scale evolved from an earlier study by our research team [39], with reference to existing literature [38,40]. Treatment adherence was an important component of the chronic illness self-management scale, which has been validated among PCHC aged 12 to 45 with confirmatory factor analysis [39]. Accordingly, the scale maintained adequate fit indices in subgroup consistency validation, and had adequate results in concurrent validity testing [39]. Treatment adherence was measured by six items that ask the participants about the frequency of following situations they experienced in the past month. Sample items included “Stopping the medications or treatment on your own without consulting a doctor” and “Forgetting or skipping your medications or treatment.” It has been a typical practice to design the items to assess treatment adherence in a reversed way [38], and this study also adopted this approach. Higher scores indicated better treatment adherence. The reliability of treatment adherence was 0.886 in the baseline survey and 0.890 in the follow-up survey.

Supportive interaction focuses on the depth and variation of interaction or communication among members of mutual aid groups, which may influence the attitude and behavior of the entire group [17]. With reference to the existing literature [21,26], supportive interaction was measured by six items by asking the participants how often the group members (including the participant themselves) had performed the following actions in the past six months. Sample items were “Group members encourage the others to express their opinions”, “Group members interrupt the others while they are talking”, and “Group members take the initiative to make comments”. Higher scores revealed higher levels of supportive interaction in mutual aid groups. The reliability of supportive interaction was 0.961 in the baseline survey and 0.961 in the follow-up survey.

Facilitator input variety refers to the number of different topics that were raised and discussed by facilitators in mutual aid groups to provide support for issues faced by young PCHC in different areas of their lives [21]. To measure this, nine items on a dichotomous (yes or no) level were constructed asking the participants if the facilitator(s) had helped their groups with issues pertaining to different life aspects including work, education, finance, physical health, and family in the past six months. Higher scores reflected a wider variety of facilitator’s input in the groups. The reliability of facilitator input variety was 0.761 in the baseline survey and 0.758 in the follow-up survey.

### 2.4. Controlling for Background Factors

Extant studies have shown that various background factors, such as gender, family income, age, and education level, are also associated with treatment adherence (e.g., [43]). For example, poor treatment adherence might be more likely seen among younger patients and patients with a low education level and low family income [44]. There are also gender differences in treatment adherence; for instance, females are more likely to take their medicine on time than males [45]. In addition, illness category is a possible influential factor for treatment adherence because some psychosocial factors, such as social stigma and insufficient social support related to different illnesses, may further complicate treatment adherence [46]. Previous studies reported that facilitator background could influence treatment adherence of mutual aid group members (e.g., [20]) as facilitators may perform differently due to differences in the depth and breadth of their experiential and professional knowledge [47]. In this study, these key background variables, including gender, monthly family income, age, educational level, illness category, number of professional facilitators in a mutual aid group, and number of nonprofessional facilitators in a mutual aid group, were all treated as control variables to rule out their potential influences.

## 3. Results

Structural equation modeling (SEM) was conducted using Mplus software to test the hypotheses in this study [48]. This study assessed the goodness of fit using four model-fit indices, namely root-mean-square error of approximation (RMSEA), comparative goodness-of-fit index (CFI), Tucker–Lewis Index (TLI), and standardized root-mean-square residual (SRMR), with the exception of chi-square due to its high sensitivity to sample size [49].

Table 2 shows the results of mean comparisons of data between the baseline and follow-up surveys. In the baseline survey, the average score for supportive interaction was 34.4 (*SD* = 35.0), the average score for facilitator input variety was 11.8 (*SD* = 18.6), and the average score for treatment adherence was 70.9 (*SD* = 23.0). In the follow-up survey, the average score for supportive interaction was 36.7 (*SD* = 35.4), the average score for facilitator input variety was 12.6 (*SD* = 18.9), and the average score for treatment adherence was 72.5 (*SD* = 22.8). In particular, the results showed that participants indicated higher treatment adherence, supportive interaction, and facilitator input variety in the follow-up survey than in the baseline survey.

Table 3 shows the results of the delayed effects of predictors in the baseline survey on treatment adherence in the follow-up survey. The model attained a perfect fit (see Table 3, *R*^2^ = 0.470, RMSEA = 0, CFI = 1, TLI = 1, and SRMR = 0), as it was a saturated model. According to the results, supportive interaction and facilitator input variety in the baseline survey each had significant positive effects on treatment adherence in the follow-up survey (β = 0.156 and β = 0.098), with controls for treatment adherence in the baseline survey and background factors, supporting Hypotheses 1 and 2.

To examine the immediate effects of supportive interaction and facilitator input variety on treatment adherence, the modeling included supportive interaction and facilitator input variety in the follow-up survey for predicting treatment adherence in the follow-up survey for further analysis, controlling for background factors. This saturated model also had a perfect fit (see Table 4, *R*^2^ = 0.471, RMSEA = 0, CFI = 1, TLI = 1, and SRMR = 0). The results demonstrated that after adding the two predictor variables from the follow-up survey, supportive interaction in the baseline survey had a marginally significant effect on treatment adherence in the follow-up survey (β = 0.155), whereas facilitator input variety in the baseline survey did not show a significant effect on treatment adherence in the follow-up survey. More important, supportive interaction and facilitator input variety in the follow-up survey did not have a significant effect on treatment adherence in the follow-up survey. In other words, supportive interaction and facilitator input variety did not manifest significant immediate effects on the treatment adherence of young PCHC. The findings thus gave no support to Hypotheses 3 and 4.

Regarding the reversed effects, treatment adherence in the baseline survey showed a significant effect on supportive interaction in the follow-up survey (see Table 4, β = 0.088), supporting Hypothesis 5. Nevertheless, treatment adherence in the baseline survey did not show a significant effect on facilitator input variety in the follow-up survey (β = −0.009), rejecting Hypothesis 6. Moreover, the results found significant relationships between the two predictor variables in the baseline and follow-up surveys (see Table 4). These findings suggested that supportive interaction in the baseline survey had a significant effect on facilitator input variety in the follow-up survey (β = 0.221), while facilitator input variety in the baseline survey also had a significant effect on supportive interaction in the follow-up survey (β = 0.142), supporting Hypotheses 7 and 8. These two models were saturated models with a perfect fit (see Table 4, *R*^2^ = 0.635 and 0.527, RMSEA = 0, CFI = 1, TLI = 1, and SRMR = 0).

In addition, the results also showed that female participants tend to receive less diverse facilitator input (β = −0.115), while participants with higher educational levels and mental illnesses are more likely to receive a wider variety of facilitator input in mutual aid groups (β = 0.162 and β = 0.118, respectively). Moreover, the number of nonprofessional facilitators in mutual aid groups was also found to have a significant effect on facilitator input variety (β = 0.105).

## 4. Discussion

Although extant studies have pointed out that time is a key factor influencing the outcomes of mutual aid groups (e.g., [18]), little research has been done on the time effects of supportive interaction and facilitator input variety on treatment adherence, especially in the context of young PCHC [17]. To fill the gap, this study investigated the immediate and delayed effects of supportive interaction and facilitator input variety on treatment adherence of young PCHC and possible reversed effects among the three variables. Specifically, in this study, delayed effects are the effects of supportive interaction and facilitator input variety (i.e., the predictor variables) in the baseline survey on treatment adherence in the follow-up survey, whereas immediate effects refer to the instant effects of supportive interaction and facilitator input variety on treatment adherence of group members, all of which were measured in the follow-up survey.

In general, the results of this study are consistent with previous findings [15,50] that show supportive interaction and facilitator input variety have positive outcomes for participants in mutual aid groups [14,17,51]. Specifically, supportive interaction has been viewed as a co-creation process of rehabilitation [52], through which members of mutual aid groups utilize each other’s emotional and informational support [50]. As supportive interaction ensures frequent information sharing and reciprocal exchange of resources among group members [16], this interactive dynamic consequently helps members of the groups achieve improvements in their treatment adherence [18,52]. Additionally, this study provides strong evidence that facilitator input variety of mutual aid groups also plays a critical role in the rehabilitation journey of young PCHC. The results of this study suggested that a more holistic and comprehensive approach to facilitator input could lead to better treatment adherence of young PCHC [20], as members in mutual aid groups often face a wide range of difficulties in different areas of their lives.

Importantly, by introducing a time perspective, the findings of this study add to the existing literature dealing with the effectiveness of mutual aid groups. Although previous research has provided support for the positive effects of supportive interaction and facilitator input variety on treatment adherence (e.g., [15,16]), the time effects of such influences have largely been neglected. As time is a key factor in mutual aid groups [26,29], this study proposed eight hypotheses concerning the delayed and immediate effects of supportive interaction and facilitator input variety on treatment adherence, as well as the reversed effects of treatment adherence on supportive interaction and facilitator input variety, the reserved effect of supportive interaction on facilitator input variety, and the reversed effect of facilitator input variety on supportive interaction. Notably, five of the eight hypotheses proposed in this study were supported by statistically significant findings. For the delayed effects, the results revealed that participants experienced improvements in treatment adherence in the follow-up survey, resulting from supportive interaction and facilitator input variety in the baseline survey. On the other hand, the results showed nonsignificant immediate effects of supportive interaction and facilitator input variety on treatment adherence of young PCHC. In other words, the findings of this study suggest that the positive effects of supportive interaction and facilitator input variety exhibit their impacts primarily in the delayed situation, which supports the idea that engagement in mutual aid groups is a relatively longer-term rehabilitation process and its impacts take time to develop [18,29]. Moreover, the nonsignificant immediate effects may also be explained by the cultural differences. In Chinese culture, chronic health conditions are often associated with negative feelings and patients may perceive actively asking help from others as shameful [53]. Chinese culture also puts a great emphasis on self-suppression so that people’s emotional distress and physical symptoms are often hidden [6]. These negative views and cultural beliefs could hinder the progress of young PCHC in trust-building and behavior-changing in the groups, as group members might be reluctant to talk about their illness with others, express their physical and emotional discomfort, and seek support from other members [54]. As a result, it might take a longer time for young PCHC to benefit from supportive interaction and facilitator input variety in mutual aid groups in a Chinese context. Despite taking longer time for PCHC with a Chinese cultural background to talk about their illness and express their thoughts in mutual aid groups, they eventually demonstrate improvements as reflected by the significant delayed effects in this study.

For the reversed effects, this study identified a reverse relationship between treatment adherence and supportive interaction, in which treatment adherence in the baseline survey was found to have a significant positive effect on supportive interaction of young PCHC in the follow-up survey. One possible explanation is that people with better treatment adherence are often associated with symptom improvement [55], and thus might have a higher motivation to engage in supportive interaction within mutual aid groups and actively contribute to the groups by working on other members’ problems. However, the results of this study showed a nonsignificant reversed effect of treatment adherence on facilitator input variety. This might be because the variety of facilitator input largely depends on each facilitator’s own expertise and prior experience [20], and the content of the mutual aid groups is normally discussed and decided on with the participants based on their actual needs [9]. Thus, the treatment adherence of an individual member may have limited influence on the facilitator input variety.

In addition, this study found significant relationships between supportive interaction and facilitator input variety in the baseline and follow-up surveys. The results showed that young PCHC with a higher level of supportive interaction in the baseline survey tend to receive a wider variety of facilitator input in the follow-up survey, and participants receiving more diverse facilitator input in the baseline survey are more likely to have a higher level of supportive interaction in the follow-up survey. The explanation here might be that members who have a higher level of supportive interaction tend to feel more comfortable in expressing their feelings and seeking mutual support in mutual aid groups [14,30], leading them to take the initiative to interact with facilitators and seek advice on different areas of their lives. Similarly, group members who receive a wider variety of facilitator input might have a better knowledge of chronic illness management and feel more empowered [20], which enables them to actively engage in group meetings to share with other members and work on others’ problems [9,56].

Moreover, several background characteristics have been controlled in this study. The outcomes denote that facilitator input variety in mutual aid groups was influenced by four background factors: gender, education, mental illnesses, and the number of nonprofessional facilitators in the mutual aid groups. First, the results showed that female participants seemed to receive less diverse facilitator input. This might associate with their perceptions of illness. Past studies found that women tend to value higher personal control in managing their illness [45] and are more sensitive to the problems related to their chronic health conditions [57]. Therefore, female participants may take the initiative to talk about the issues concerning them in group meetings without demanding much facilitator involvement. Additionally, the level of education was found to be positively associated with facilitator input variety. This is consistent with previous studies suggesting that patients with higher educational levels are usually more concerned about their chronic health conditions and treatment [58], which may motivate them to actively seek support and advice from facilitators on different areas related to illness management. This study also found that patients with mental illnesses received more diverse facilitator input. This might be because mental illnesses can affect many areas of patients’ lives and these patients need extra support and help to navigate challenges [43], leading to extra effort and diverse input from facilitators in mutual aid groups. In addition, the results also showed that the number of nonprofessional facilitators in a mutual aid group was positively associated with facilitator input variety, perhaps because nonprofessional facilitators mainly acquired their knowledge and skills from their hands-on experience in managing chronic health conditions [20]. As chronic illness-related problems are multidimensional, nonprofessional facilitators may have diverse experience and approaches in their illness management covering different aspects of their lives, which consequently could lead to a wider variety of facilitator input in mutual aid groups.

Despite the evidence for important time effects of supportive interaction and facilitator input variety in mutual aid groups on treatment adherence of young PCHC, this study poses several limitations. First, this study employed the self-report method. Although self-report measures have been popular in the literature, this method may be subject to social desirability bias and recall bias [59]. A combination of different measurements such as medical records, information from family members, or medication refill rates should be collected in future studies to address the limitations of self-report. Second, this study discussed people with ten specific types of chronic health conditions, all having their own distinctive patterns and impacts. Future studies are necessary to investigate the dynamic among supportive interactions, facilitator input variety, and treatment adherence of people living with other chronic health conditions. Third, this study focused on young PCHC in Hong Kong, a city whose unique cultural and healthcare context might have influenced the results (e.g., [6,53,54]). To ensure the generalization of the research results, further studies may consider adopting a comparative approach and illustrating the cross-context applicability of the results. Results from different contexts would better guide theoretical explorations and practices. Fourth, this study collected data at baseline and follow-up surveys a year apart. Future research could collect intermediate measurements to examine whether change happens at an earlier time.

Nevertheless, this study contributes to the literature in various ways and has important implications for practices. First, this study enriches the existing literature with a new perspective by focusing on young PCHC aged between 12 and 45 years [40]. Considering the early onset of chronic health conditions, the findings of this study provide an important understanding of how the participation in mutual aid groups could enhance treatment adherence of young PCHC [17]. Furthermore, this study provides empirical evidence for the importance of supportive interaction and facilitator input variety in improving treatment adherence in mutual aid group settings [15,16]. This study advanced prior studies by exploring the dynamic mechanism of mutual aid groups using a time perspective in which the effects of supportive interaction and facilitator input variety on treatment adherence of the group members are time-sensitive [18,29].

In addition, the findings also provide important insights to practitioners. The results of this study confirm that the engagement in supportive interaction and facilitator input in mutual aid groups are important factors in enhancing treatment adherence of young PCHC [14,15]. Young PCHC can benefit from active communication and exchange of mutual support with group members [18] and from a more holistic and comprehensive approach adopted by the facilitator in assisting mutual aid groups [20]. Practitioners should consider encouraging facilitators to cover more diverse topics in group meetings and use different strategies to facilitate active engagement of group members for better rehabilitation outcomes. In addition, it is important to note that the effects of the two predictor variables are observable only after a delay [28]. As shown in our study, an immediate measurement approach, such as using a post-session survey right after the intervention, may not apply to the evaluation of mutual aid groups as the impact on the groups often needs time to develop and manifest [29]. Thus, further evaluation measures at different times are necessary. Moreover, this study uncovered the underlying mechanism of how treatment adherence of young PCHC and supportive interaction in mutual aid groups reinforce each other over time [32]. In light of this new finding, practitioners may consider integrating participants with different levels of treatment adherence in a mutual aid group to facilitate such a virtuous cycle for promoting better rehabilitation outcomes.

## 5. Conclusions

Taking the time effects into consideration, we investigated the mechanisms by which supportive interaction and facilitator input variety, as predictor variables, in mutual aid groups impact treatment adherence, as the outcome variable, among young PCHC 12 to 45 years old.

Among the results, significant delayed effects occurred in how supportive interaction and facilitator input variety at the baseline survey predicted treatment adherence at the follow-up survey, whereas no significant immediate effects did. A significant reverse relationship also emerged, such that a higher level of treatment adherence at baseline predicted a higher level of supportive interaction at follow-up. Beyond that, the results also included significant relationships between supportive interaction and facilitator input variety in the baseline and follow-up surveys. Lastly, four background factors significantly influenced facilitator input variety. In particular, female participants experienced lower facilitator input variety, while participants with a higher level of education, with mental illnesses, or in mutual aid groups with more nonprofessional facilitators experienced higher facilitator input variety.

Taken together, the results underscore the importance of supportive interaction and facilitator input variety in mutual aid groups in efforts to enhance long-term treatment adherence among young PCHC. Those findings demonstrate that it takes time for rehabilitation processes involving supportive interaction and facilitator input variety to achieve positive effects on treatment adherence among members of mutual aid groups. Furthermore, the significant reversed effect and the significant relationships between the predictor variables suggest that the three variables can enhance one another, thereby forming a virtuous cycle for promoting better rehabilitation outcomes. Findings concerning the background factors also provide insights into the unique patterns and needs of group members with different characteristics.

## Figures and Tables

**Table 1:** Background characteristics of study participants (*N* = 391).

Characteristic	%	
Gender		
Female	55.1	
Male	44.9	
Monthly family income		
HK$4999 (US$644) or below	7.7	
HK$5000–9999 (US$645–1289)	6.7	
HK$10,000–19,999 (US$1290–2580)	13.4	
HK$20,000–29,999 (US$2581–3870)	22.2	
HK$30,000–39,999 (US$3871–5160)	25.9	
HK$40,000–49,999 (US$5161–6450)	12.3	
HK$50,000 (US$6451) or above	11.8	
Educational level		
Primary (grade 6) or below	3.5	
Secondary (grades 7–12)	40.9	
Higher diploma or associate degree	14.7	
Bachelor’s degree	37.0	
Master’s degree or above	3.9	
Illness category		
Asthma	5.0	
Heart disease	3.1	
Diabetes	5.5	
Rheumatic diseases	17.5	
Neurological diseases	10.7	
Hematologic diseases	6.5	
Cancer	5.2	
Rare diseases	4.7	
Eczema	18.8	
Mental illnesses	23.0	
**Characteristic**	**Mean**	***SD***
Age (years)	30.2	7.7
Number of professional facilitators (persons)	1.6	2.0
Number of nonprofessional facilitators (persons)	0.9	1.9

**Table 2:** Means and standard deviations (*N* = 391).

Variables	Scoring	Mean	*SD*
Treatment adherence (follow-up)	0–100	72.5	22.8
Supportive interaction (follow-up)	0–100	36.7	35.4
Facilitator input variety (follow-up)	0–100	12.6	18.9
Treatment adherence (baseline)	0–100	70.9	23.0
Supportive interaction (baseline)	0–100	34.4	35.0
Facilitator input variety (baseline)	0–100	11.8	18.6

**Table 3:** Standardized effects of the baseline survey predictors.

Predictors	Treatment Adherence (Follow-up)
Treatment adherence (baseline)	0.577 ***
Supportive interaction (baseline)	0.156 **
Facilitator input variety (baseline)	0.098 *
Female	0.019
Age	−0.037
Education	−0.006
Monthly family income	0.003
Mental illnesses	−0.022
Number of professional facilitators	−0.029
Number of nonprofessional facilitators	−0.035
*R* ^2^	0.470

Note. Estimates from the saturated model by robust maximum likelihood estimation in Mplus. * *p* < 0.05, ** *p* < 0.01, *** *p* < 0.001.

**Table 4:** Standardized effects of the predictors in the baseline and follow-up surveys.

Predictors	Treatment Adherence (Follow-up)	Supportive Interaction (Follow-up)	Facilitator Input Variety (Follow-up)
Supportive interaction (follow-up)	−0.011		
Facilitator input variety (follow-up)	0.036		
Treatment adherence (baseline)	0.578 ***	0.088 *	−0.009
Supportive interaction (baseline)	0.155 +	0.651 ***	0.221 ***
Facilitator input variety (baseline)	0.082	0.142 ***	0.490 ***
Female	0.024	0.011	−0.115 **
Age	−0.039	−0.038	0.031
Education	−0.011	0.042	0.162 ***
Monthly family income	0.005	−0.008	−0.066
Mental illnesses	−0.027	−0.037	0.118 **
Number of professional facilitators	−0.032	0.088	0.101
Number of nonprofessional facilitators	−0.039	−0.005	0.105 ***
*R* ^2^	0.471	0.635	0.527

Note. Estimates from the saturated model by robust maximum likelihood estimation in Mplus. + *p* < 0.10, * *p* < 0.05, ** *p* < 0.01, *** *p* < 0.001.

## Data Availability

The datasets generated during and/or analyzed during the current study are not publicly available due to datasets containing information that could compromise the privacy of research participants. The data that support the findings of this study are available from the corresponding author (S.S.-y.N.) upon reasonable request.
